# Rotating Scheimpflug Imaging Indices in Different Grades of Keratoconus

**DOI:** 10.1155/2016/6392472

**Published:** 2016-08-08

**Authors:** Sherine S. Wahba, Maged M. Roshdy, Rania S. Elkitkat, Karim M. Naguib

**Affiliations:** ^1^Faculty of Medicine, Ain Shams University, Cairo 11566, Egypt; ^2^Al Watany Eye Hospitals, 211 Alhegaz Street, Alhegaz Square, Heliopolis, Cairo 11361, Egypt

## Abstract

*Purpose*. To evaluate accuracy of various Keratoconus (KC) screening indices, in relation to Topographic Keratoconus (TKC) grading.* Setting*. Al Watany Eye Hospital, Cairo, Egypt.* Methods. *Data of 103 normal (*group 1*) and 73 KC eyes (*group 2*), imaged by Pentacam (branded as Allegro Oculyzer), were analysed.* Group 2* was divided into* 2a*: 14 eyes (TKC = 1, early KC),* 2b*: 25 eyes (TKC = 1 to 2 or 2, moderate KC), and* 2c*: 34 eyes (TKC = 2 to 3 up to 4, severe KC). Participants were followed up for six years to confirm diagnosis. Area under the receiver operating characteristic curve (AUROC) was calculated for evaluated curvature, elevation, and pachymetry indices with various reference shapes at different diameters.* Results*. When comparing normal to KC eyes, ten indices had significantly higher AUROC. Only five of them had significantly higher AUROC in early KC compared to normal corneas: Pachymetry Progression Index- (PPI-) Maximum (Max), Ambrósio's Relational Thickness- (ART-) Max, PPI-Max minus PPI-Minimum (Min), central corneal thickness (CCT), and diagonal decentration of thinnest point from the apex (AUROC = 0.690, 0.690, 0.687, 0.683, and 0.674, resp.).* Conclusion*. Generally, ten pachymetry and elevation-based indices had significantly higher AUROC. Five indices had statistically significant high AUROC when comparing early KC to normal corneas.

## 1. Introduction

The hallmark characteristic of Keratoconus (KC) is the development of localized, cone-shaped ectasia accompanied by corneal thinning in the area of the cone. This leads to irregular astigmatism and steeper corneal curvature over the area of the cone [1]. The early detection of these changes is usually based on Placido-based corneal topography and rotating Scheimpflug camera corneal tomography. However, there are confounding conditions that can imply topographic and tomographic corneal changes resembling KC, such as corneal scarring, hyperopic refractive surgery, contact lens-induced warpage [2], and even normal variants of topographic curvature patterns.

There are several tomographic criteria for KC diagnosis. They can be divided into three main subgroups: curvature-based, elevation-based, and pachymetry-based. Unfortunately, none of them is 100% sensitive and specific. Some authors believe that elevation maps are better than axial curvature maps for KC screening [3]. Others claim that curvature is still the most sensitive [4].

The rotating Scheimpflug camera “Pentacam” (Oculus GmbH, Wetzlar, Germany) can generate various indices within each of the three indices subgroups.

Pentacam allows for measuring local elevation points by fitting the corneal shape to a best-fit sphere (BFS) reference surface with variable diameters or to a best-fit toric ellipsoid surface (BFTE), which simulates more the corneal shape than the BFS [5]. The guidelines differ between authorities. For instance, some prefer using BFS based on the analysis of 8 mm zone around the corneal apex, especially for refractive surgery screening [6, 7], others keep the device default at 9 mm zone, although there are some concerns about it; that is, it is more difficult to get reliable scans without wide palpebral fissure. On the contrary, BFTE has been recommended for use by other studies, as they concluded that toric ellipsoid reference surface is the most sensitive reference body to compare KC to normal corneas [8–10].

More recently, there have been other proposed indices; one of the most valuable KC indices is the Ambrósio's Relational Thickness (ART) [11].

This study aims at comparing the accuracy of each of the tested indices at various best-fit reference surfaces, in cohorts of KC and normal corneal cases who were followed up for six years, and to evaluate the accuracy of various indices in relation to Topographic KC (TKC) grading.

To the best of our knowledge, our study is innovative in evaluating the accuracy of KC indices using different reference surfaces of various diameters when applicable. Moreover, novelty lies in comparing the tested indices against time evidence, through our six-year follow-up interval (stability even after LASIK surgeries of normal corneas and spontaneous progression over years in KC cases). Time evidence is a definite proof of diagnosis not relying on subjective assessment.

## 2. Patients and Methods

A retrospective study was conducted using the data from consecutive patients' files that were imaged in the time interval between June 2008 and December 2009, using the Pentacam branded as Allegro Oculyzer (WaveLight, GmbH, Erlangen, Germany) [12] with software version 1.16r12 at Al Watany (National) Eye Hospital, Cairo, Egypt. Patients with any detected corneal pathology other than KC and patients with history of ocular surgery, contact lens wear during the previous two weeks, or narrow palpebral fissure impeding reliable imaging were excluded from the study. Any patient with questionable diagnosis at the time of initial Pentacam imaging was also excluded. Moreover, the patients were followed up until December 2015 to confirm the diagnosis and hence to confirm and evaluate the results.

Every eye was scanned at least thrice by the Allegro Oculyzer, in a dark room and according to the recommendations stated in the device manual. Each scan included 25 Scheimpflug images. Despite good repeatability, data were collected from the most reliable scan as stated by the “QS” pop-up box (i.e., largest analysed area, valid data percent, and good alignment). The data were collected from the automatically calculated indices or generated by manually changing the reference surface shape (BFS or BFTE) and calculation area (7, 8, or 9 mm) and getting the elevation values on mouse click. The investigated indices were as follows.

Curvature indices are as follows:

- Mean eccentricity of the anterior corneal surface in the central 3 mm.
- Mean radii of curvature of anterior and posterior corneal surfaces in the central 3 mm (in mm).
- Determinants of BFS:
  - radii of curvature of the 8 and 9 mm anterior BFS (in mm),
  - radii of curvature of the 7, 8, and 9 mm posterior BFS (in mm).
- Determinants of posterior BFTE:
  - eccentricity of the 7, 8, and 9 mm posterior BFTE,
  - flattest and steepest radii of curvature of the 7, 8, and 9 mm posterior BFTE (in mm).

Elevation indices are as follows:

- Elevation of the thinnest point from the 7 mm posterior (PE) BFS (in *μ*m).
- Elevation of the thinnest point from the 8 and 9 mm anterior (AE) and posterior (PE) BFS and difference between corresponding anterior and posterior values (in *μ*m).
- Elevation of the thinnest point from the 7, 8, and 9 mm posterior (PE) BFTE (in *μ*m).
- Maximum elevation, minimum elevation, and the difference between them (in *μ*m) from the 8 and 9 mm anterior BFS.
- Maximum elevation, minimum elevation, and the difference between them (in *μ*m) from the 7, 8 and 9 mm posterior BFS.
- Maximum elevation, minimum elevation, and the difference between them (in *μ*m) from the 7, 8, and 9 mm posterior BFTE.

Pachymetry indices are as follows:

(i) Pachymetry at corneal apex, corneal center, and thinnest point (in *μ*m).
(ii) Pachymetry Progression Index-Average (PPI-Avg), Minimum (PPI-Min), and Maximum (PPI-Max) and the difference between PPI-Max and PPI-Min (PPI-Max minus PPI-Min).
(iii) Ambrósio's Relational Thickness-Average (ART-Avg), Minimum (ART-Min), and Maximum (ART-Max).
(iv) Horizontal and vertical decentration of the thinnest point from the apex and the resultant diagonal decentration [=√(squared horizontal decentration + squared vertical decentration) (in mm)].

Patients were classified into two groups according to TKC staging adopted by the device software;* group 1* included 103 normal eyes, while* group 2* is comprised of 73 eyes with KC. Group 2 was furtherly divided into subgroups:* group 2a*: 14 eyes (TKC = 1, early KC),* group 2b*: 25 eyes (TKC = 1 to 2 or 2, moderate KC), and* group 2c*: 34 eyes (TKC = 2 to 3, 3, 3 to 4, or 4, severe KC).

The study adhered to the Tenets of the Declaration of Helsinki and to the local ethics standards.

### 2.1. Statistical Analysis

Data were collected and verified, and the compound indices were calculated using Microsoft Excel 2010 (Redmond, Washington, USA). Statistical analyses were performed using IBM SPSS Statistics (v19; Armonk, NY, USA) and Medcalc v11.1.1.0 (MedCalc, Belgium).

The following tests were performed: calculation of the mean, standard deviation (SD), unpaired *t*-test, sensitivity, specificity at different cut-off values, likelihood ratios, and area under the receiver operating characteristic curve (AUROC). AUROCs are compared using the DeLong method [13]. Values were considered statistically significant if *P* value was less than 0.05.

## 3. Results

### 3.1. Cases Characteristics

Average patients' age at the time of the initial Pentacam imaging was 29.2 ± 8.8 years (range 17.4 to 53.2) and 27.8 ± 7.3 years (range 14 to 44.4) in normal and KC groups, respectively. There was no statistically significant difference between the two groups (*P* = 0.253). The ratios between right and left eyes were 53 : 50 and 40 : 33 in normal and KC groups, respectively (*P* = 0.777).

The mean, SD, and 95% confidence interval (CI) of each parameter are listed in Table 1.

Table 2 represents the AUROC of all tested indices when comparing normal corneas to all other grades of KC collectively, and Table 3 represents the ten indices with the highest AUROC. The AUROCs of these ten indices were statistically noninferior to each other (*P* > 0.05). Meanwhile, all other indices had statistically inferior AUROC to that of at least one of these ten indices. It is worth mentioning that none of the curvature indices was among these ten indices.

Among the examined patients of both groups, pachymetry progression indices showed the highest AUROC accuracy among the evaluated parameters, where the Pachymetry Progression Index-Maximum (PPI-Max) and Ambrósio's Relational Thickness-Maximum (ART-Max) had the same highest AUROC (0.987). The best cut-off for PPI-Max was (>1.4 with sensitivity 91.78% and specificity 98.06%) and for ART-Max was (≤412 with sensitivity 97.26% and specificity 93.2%).

The further analysis of the indices according to patients' grouping (based on TKC grading) showed that the mean values of all indices were significantly different when comparing group 2c corneas and normal corneas (*P* < 0.05), with the only exception of horizontal decentration of the thinnest point from the apex (*P* = 0.292). Meanwhile, only three indices were statistically insignificant when comparing their values in group 2b keratoconic corneas against group 1 normal corneas (radius of curvature of the 9 mm posterior BFS (*P* = 0.061) and maximum elevated point of the 9 mm posterior BFTE (*P* = 0.361), in addition to horizontal decentration of the thinnest point from the apex (*P* = 0.150). Contrarily, only five indices' values were statistically significant in discriminating group 2a KC corneas from normal corneas, as shown in Table 4.

## 4. Discussion

Placido-based systems rely on the analysis of a reflected image. This cannot provide data from the posterior corneal surface [6]. Additionally, without information about the posterior corneal surface, complete pachymetric evaluation is not possible [14]. Ultrasonic central pachymetry measures one point, which is not necessarily the thinnest point, and does not reflect the overall thickness profile [15]. Moreover, the posterior corneal surface is appreciated as a sensitive indicator of corneal ectasia and can often be abnormal in spite of a still normal anterior corneal surface. While the corneal refractive power is largely determined by the anterior surface, the evaluation of anatomical properties of the cornea is at least equally dependent upon both surfaces [14]. Therefore, corneal tomography parameters assessment appeared to be essential in detecting KC.

With the appearance of many KC indices and suggestion of various best-fit reference surfaces [6], the goal of our study was to evaluate the accuracy (including both sensitivity and specificity) of such indices with the use of different reference surfaces, at different diameters, and correlate these parameters to TKC grading. Some previous studies used cyclic evidence that stated that a certain index was accurate based on differentiating KC from normal cornea using certain criteria closely related to this same index. Our study adds the long follow-up (6 years) as a more robust evidence. If a cornea was falsely diagnosed as normal and underwent LASIK, it would develop ectasia in such a long follow-up period. On the other side, a KC diagnosis can be confirmed if it spontaneously progressed over time.

The inclusion of every single possible index is out of the scope of this study. The included indices are those mentioned individually in other studies or those we thought they might be significant. On evaluating BFTE and smaller best-fit surface (7 mm), we focused on the posterior rather than the anterior corneal surface, being the site for primary subclinical tomographic changes, preceding the anterior surface [16–19].

Curvature-based indices derived from both anterior and posterior surfaces were evaluated and analysed. According to the ROC curves, all evaluated curvature indices had statistically significant less AUROC for diagnosing KC than most of elevation and pachymetry indices. Previous reports demonstrated similar results [20–22]. Despite being the least significant in diagnosing KC, they have been considered as important criteria in KC and after LASIK ectasia follow-up [23, 24].

In this study, where we only analysed data from good quality scans with corneal surface area more than 9 mm, analysis of elevation indices showed that posterior surface elevation (PE) from 8 mm BFS and anterior surface elevation (AE) from 9 mm BFS had the highest accuracy, with AUROC (0.979) for both, followed by PE from 7 and 9 mm BFS (AUROC = 0.977 and 0.971, resp.). AE and PE from reference best-fit surfaces did not differ in their accuracy with various diameters (7, 8, and 9 mm). Correia et al. reported that PE from both 8 mm BFS and BFTE had highest AUROC (0.983 and 0.986, resp.) [20]. de Sanctis et al. [25] evaluated the sensitivity and specificity of PE from 9 mm BFS in discriminating normal corneas from KC. The AUROC analyses showed high overall predictive accuracy of PE for KC (AUROC 0.99). They concluded that PE very effectively discriminates KC from normal corneas, but its efficacy is lower for subclinical KC, and thus data concerning PE should not be used alone to stratify patients with this condition.

We enrolled in our work the elevation indices of the thinnest point from both BFS and BFTE. The latter was evaluated in literature and showed higher predictive accuracy in diagnosing KC and forme fruste KC compared to BFS, especially with a fixed eccentricity. A possible explanation for its diagnostic superiority in ectatic corneas is that its central vaulting can determine whether the corneal pattern is associated with an atypical condition, a true corneal disease, or an artifact of alignment or processing [16, 20].

According to our study results, parameters that had the highest AUROC for diagnosing KC were the PPI-Max at cut-off value of >1.4 and the ART-Max at cut-off value of ≤412 (0.987 for both). These results were comparable to those of Ambrósio Jr. et al. [1] who reported that the most sensitive parameters were ART-Avg and ART-Max (AUROC = 0.987 and 0.983, resp.).

Our study investigated the accuracy of various tomographic indices in relation to TKC grading [26]. We found that most of the tested indices had significantly different values between each of grades 2 to 4 (moderate and/or severe) KC and normal corneas. In 2012, Ishii and his coworkers [27] investigated the severity of KC in terms of corneal elevation differences, and they correlated the data with Amsler-Krumeich classification of KC. They observed that cases of higher elevation differences in the anterior and posterior corneal surfaces were staged higher in Amsler-Krumeich classification (*P* < 0.001) and concluded that anterior and posterior corneal surface height data obtained by elevation-based tomography provide useful information in improving KC diagnostic accuracy and in grading the severity of KC. Their results are comparable to ours in the accuracy of elevation indices; however, they did not furtherly investigate other indices.

Furthermore, Kamiya and his colleagues [28] evaluated corneal elevation, pachymetry, and keratometry in KC eyes according to the clinical stage of the disease, with respect to each stage of Amsler-Krumeich classifications. They detected that PE (0.980) and AE (0.977) showed the highest AUROCs, followed by *K* max value (0.941), percentage thickness increase (PTI) 2 mm (0.931), PTI 4 mm (0.927), and PPI-Max (0.927). Their results are highly comparable to ours in AUROC of indices. They observed that AE and PE measurements tended to have a higher accuracy at the earlier stages of KC, when compared to other studied parameters; hence, they concluded that elevation difference measurements might provide useful information for improving the diagnostic accuracy of KC, especially in the early stage. These results contradict ours, where we found that the indices with statistically significant high AUROC when comparing grade 1 KC to normal corneas were PPI-Max, ART-Max, PPI-Max minus PPI-Min, central corneal thickness, and diagonal decentration of the thinnest point from the apex.

Our findings justify the modifications in the newer software versions that incorporated “ART-Max” and “diagonal decentration of the thinnest point from the apex,” being sensitive indices for early KC detection. As regards the hardware changes, we also recommend similar studies on Pentacam HR, because the current one was on Allegro Oculyzer I Pentacam. Randleman et al. [29] reported that, for refractive surgical screening, regular and high-resolution Scheimpflug imaging devices generated different objective values and the two devices are not interchangeable.

## 5. Conclusion

The accuracy of different curvature, elevation, and pachymetry-based indices using various reference shapes of different diameters was evaluated, in relation to TKC grading. To the best of our knowledge, this is the first study to confirm clinical and topographical observations by a long follow-up period of 6 years to ascertain the diagnosis by time evidence. Generally, to discriminate all KC grades from normal, ten pachymetry and elevation-based indices were significantly more accurate than other indices, having higher AUROC. Five of them had statistically significant high AUROC when comparing early KC to normal corneas.

## Figures and Tables

**Table 1:** The mean, SD, and 95% confidence interval (CI) of all tested KC detecting parameters. Radii of curvature are in mm, elevations in *μ*m, pachymetry in *μ*m, and decentration in mm.

Indices	Normal			Keratoconus stages 1 to 4		
	Mean	SD	95% CI	Mean	SD	95% CI
*Curvature indices*						

*Central 3 mm*						
Mean radius of curvature of anterior 3 mm corneal surface	7.725	0.2305	7.323–8.149	6.954	0.6640	5.296–8.124
Mean radius of curvature of posterior 3 mm corneal surface	6.425	0.256	6.040–6.809	5.624	0.685	4.145–6.777
Mean eccentricity of the anterior corneal surface	0.519	0.132	0.210–0.730	0.799	0.3312	−0.034–1.310
*Determinants of BFS*						
Radius of curvature of the 8 mm anterior BFS	7.786	0.2252	7.413–8.200	7.350	0.445	6.191–8.230
Radius of curvature of the 9 mm anterior BFS	7.833	0.2225	7.462–8.269	7.488	0.398	6.535–8.284
Radius of curvature of the 7 mm posterior BFS	6.390	0.2147	5.991–6.758	5.935	0.4148	5.109–6.700
Radius of curvature of the 8 mm posterior BFS	6.464	0.3544	6.031–6.790	6.125	0.3842	5.465–7.022
Radius of curvature of the 9 mm posterior BFS	6.548	0.3426	6.101–6.899	6.280	0.3041	5.765–6.981
*Determinants of BFTE*						
Flattest radius of curvature of the 7 mm posterior BFTE	6.644	0.2495	6.143–7.152	5.972	0.7057	4.312–6.947
Steepest radius of curvature of the 7 mm posterior BFTE	6.175	0.2317	5.741–6.609	5.273	0.6972	3.971–6.630
Eccentricity of the 7 mm posterior BFTE	0.181	0.3173	−0.441–0.765	0.618	0.5282	−0.447–1.350
Flattest radius of curvature of the 8 mm posterior BFTE	6.655	0.2507	6.143–7.169	5.972	0.7057	4.312–6.947
Steepest radius of curvature of the 8 mm posterior BFTE	6.175	0.2317	5.741–6.609	5.278	0.6936	3.971–6.630
Eccentricity of the 8 mm posterior BFTE	0.181	0.3173	−0.441–0.765	0.618	0.5282	−0.447–1.350
Flattest radius of curvature of the 9 mm posterior BFTE	6.658	0.2665	6.143–7.169	5.972	0.7057	4.312–6.947
Steepest radius of curvature of the 9 mm posterior BFTE	6.175	0.2317	5.741–6.609	5.287	0.7092	3.971–6.630
Eccentricity of the 9 mm posterior BFTE	0.181	0.3173	−0.441–0.765	0.618	0.5282	−0.447–1.350

*Elevation indices*						

*Using BFS*						
Elevation of the thinnest point from the 7 mm posterior BFS	1.5	3.13	−4.0–9.9	46.2	32.7	2.3–115.4
Maximum elevation from the 7 mm posterior BFS	32.8	15.12	17.1–72.6	79.9	34.3	27.7–153.7
Minimum elevation from the 7 mm posterior BFS	−34.4	15.4	−75.0–−14.1	−76.5	33.5	−160.9–−29.3
Maximum minus minimum elevation from the 7 mm posterior BFS	67.2	29.2	34.0–142.8	156.48	64.2	60.6–296.0
Elevation of the thinnest point from the 8 mm anterior BFS	2.6	1.5	0.0–6.0	26.7	17.7	2.0–68.7
Elevation of the thinnest point from the 8 mm posterior BFS	4.1	4.7	−2.0–17.8	58.4	39.0	6.3–143.4
Elevation of the thinnest point from the 8 mm posterior BFS minus that of anterior BFS	1.5	4.5	−5.0–14.0	31.8	22.5	1.0–77.0
Maximum elevation from the 8 mm posterior BFS	51.4	23.3	21.0–107.9	89.6	33.3	35.0–159.4
Minimum elevation from the 8 mm posterior BFS	−43.0	18.3	−93.9–−22.1	−90.9	41.1	−198.9–−32.0
Maximum minus minimum elevation from the 8 mm posterior BFS	94.5	37.9	49.2–188.6	180.5	71.4	67.6–351.8
Elevation of the thinnest point from the 9 mm anterior BFS	4.5	2.0	1.0–9.0	34.0	21.5	6.0–90.7
Elevation of the thinnest point from the 9 mm posterior BFS	9.9	6.8	2.0–31.9	72.7	43.9	11.0–171.8
Elevation of the thinnest point from the 9 mm posterior BFS minus that of anterior BFS	5.3	6.2	−2.0–24.9	38.7	24.0	2.3–89.0
Maximum elevation from the 9 mm posterior BFS	76.5	28.16	43.1–145.9	112.836	41.9722	48.7–212.7
Minimum elevation from the 9 mm posterior BFS	−57.2	21.2	−124.0–−33.2	−104.8	61.0	−248.4–−40.3
Maximum minus minimum elevation from the 9 mm posterior BFS	133.7	45.7	77.6–284.5	217.6	89.23	87.3–428.0
*Using BFTE*						
Elevation of the thinnest point from the 7 mm posterior BFTE	3.5	2.8	−1.0–10.0	37.6	24.9	0.0–97.01
Maximum elevation from the 7 mm posterior BFTE	18.0	10.7	6.1–40.0	71.3	54.8	17.7–225.6
Minimum elevation from the 7 mm posterior BFTE	−25.8	8.6	−47.8–−12.1	−72.8	39.6	−187.4–−18.3
Maximum minus minimum elevation from the 7 mm posterior BFTE	43.8	16.3	23.0–80.3	144.1	88.3	39.3–393.1
Elevation of the thinnest point from the 8 mm posterior BFTE	4.3	3.9	−2.0–13.0	39.5	26.45	−3.0–104.0
Maximum elevation from the 8 mm posterior BFTE	43.9	22.2	11.1–114.8	103.1	84.1	23.0–360.0
Minimum elevation from the 8 mm posterior BFTE	−33.4	11.7	−62.9–−16.1	−89.9	40.9	−185.1–−29.0
Maximum minus minimum elevation from the 8 mm posterior BFTE	77.3	28.2	32.1–141.7	192.9	114.2	68.0–557.4
Elevation of the thinnest point from the 9 mm posterior BFTE	3.6	5.6	−5.0–16.9	38.7	30.8	−18.34–111.8
Maximum elevation from the 9 mm posterior BFTE	89.9	39.5	35.2–174.6	170.8	152.9	39.6–665.5
Minimum elevation from the 9 mm posterior BFTE	−42.1	15.5	−88.3–−21.1	−114.5	50.2	−217.5–−32.7
Maximum minus minimum elevation from the 9 mm posterior BFTE	132.0	43.3	65.2–231.4	285.2	177.1	107.3–859.2

*Pachymetry indices*						

Apex thickness	545.3	35.4	471.7–624.3	482.5	39.4	384.3–573.4
Central corneal thickness	545. 5	35.5	471.7–623.4	491.4	35.3	408.9–573.4
Thinnest point thickness	543.5	35.7	471.7–622.3	465.1	68.1	361.8–566.7
PPI-Min	0.543	0.1512	0.300–0.800	1.504	0.8265	0.500–3.570
PPI-Avg	0.826	0.1365	0.600–1.100	2.104	1.0454	0.900–4.967
PPI-Max	1.077	0.1716	0.800–1.400	2.800	1.3960	1.232–6.705
PPI-Max minus PPI-Min	0.534	0.189	0.300–0.992	1.296	0.763	0.400–3.835
ART-Min	1109.9	430.9	613.0–2044.2	423.3	272.5	94.0–1045.4
ART-Avg	677.6	131.4	395.7–975.2	276.6	138.2	75.7–616.9
ART-Max	517.8	90.6	326.6–697.0	207.1	100.6	54.6–427.5
Horizontal decentration of the thinnest point from the apex	0.026	0.569	−0.948–1.019	−0.087	0.574	−0.940–0.827
Vertical decentration of the thinnest point from the apex	−0.273	0.208	−0.639–0.214	−0.519	0.347	−1.394–0.110
Diagonal decentration of the thinnest point from the apex	0.618	0.240	0.202–1.088	0.799	0.288	0.366–1.525

SD: standard deviation, 95% CI: 95% confidence interval, BFS: best-fit sphere, BFTE: best-fit toric ellipsoid, PPI-Min: Pachymetry Progression Index-Minimum, PPI-Avg: Pachymetry Progression Index-Average, PPI-Max: Pachymetry Progression Index-Maximum, ART-Min: Ambrósio's Relational Thickness-Minimum, ART-Avg: Ambrósio's Relational Thickness-Average, and ART-Max: Ambrósio's Relational Thickness-Maximum.

**Table 2:** AUROC of all indices when differentiating all grades of KC collectively from normal corneas.

Indices	AUROC	SEM	95% CI
*Curvature indices*			

*Central 3 mm*			
Mean radius of curvature of anterior 3 mm	0.885	0.03	0.828 to 0.928
Mean radius of curvature of posterior 3 mm	0.882	0.0307	0.824 to 0.925
Mean eccentricity of the anterior surface	0.811	0.039	0.745 to 0.866
*Using BFS*			
Radius of curvature of the 8 mm anterior BFS	0.812	0.035	0.747 to 0.867
Radius of curvature of the 9 mm anterior BFS	0.776	0.0374	0.707 to 0.835
Radius of curvature of the 7 mm posterior BFS	0.841	0.0323	0.779 to 0.892
Radius of curvature of the 8 mm posterior BFS	0.784	0.037	0.712 to 0.856
Radius of curvature of the 9 mm posterior BFS	0.746	0.038	0.671 to 0.821
*Using BFTE*			
Flattest radius of curvature of the 7 mm posterior BFTE	0.829	0.0344	0.765 to 0.882
Steepest radius of curvature of the 7 mm posterior BFTE	0.895	0.0298	0.840 to 0.936
Eccentricity of the 7 mm posterior BFTE	0.757	0.0402	0.687 to 0.818
Flattest radius of curvature of the 8 mm posterior BFTE	0.834	0.034	0.770 to 0.885
Steepest radius of curvature of the 8 mm posterior BFTE	0.895	0.0298	0.840 to 0.936
Eccentricity of the 8 mm posterior BFTE	0.757	0.0402	0.687 to 0.818
Flattest radius of curvature of the 9 mm posterior BFTE	0.833	0.034	0.769 to 0.885
Steepest radius of curvature of the 9 mm posterior BFTE	0.884	0.0313	0.827 to 0.927
Eccentricity of the 9 mm posterior BFTE	0.757	0.0402	0.687 to 0.818

*Elevation indices*			

Elevation of the thinnest point from the 7 mm posterior BFS	0.971	0.0137	0.934 to 0.990
Maximum elevation from the 7 mm posterior BFS	0.916	0.0202	0.865 to 0.953
Minimum elevation from the 7 mm posterior BFS	0.908	0.0219	0.855 to 0.946
Maximum minus minimum elevation from the 7 mm posterior BFS	0.923	0.0193	0.873 to 0.957
Elevation of the thinnest point from the 7 mm posterior BFTE	0.953	0.0214	0.910 to 0.979
Maximum elevation from the 7 mm posterior BFTE	0.944	0.0161	0.899 to 0.973
Minimum elevation from the 7 mm posterior BFTE	0.91	0.0264	0.858 to 0.948
Maximum minus minimum elevation from the 7 mm posterior BFTE	0.949	0.0174	0.905 to 0.976
Elevation of the thinnest point from the 8 mm anterior BFS	0.968	0.018	0.930 to 0.989
Elevation of the thinnest point from the 8 mm posterior BFS	0.979	0.00837	0.945 to 0.995
Maximum elevation from the 8 mm posterior BFS	0.837	0.0304	0.775 to 0.889
Minimum elevation from the 8 mm posterior BFS	0.89	0.0255	0.834 to 0.932
Maximum minus minimum elevation from the 8 mm posterior BFS	0.875	0.0272	0.817 to 0.920
Elevation of the thinnest point from the 8 mm anterior BFS minus that of posterior BFS	0.961	0.0139	0.920 to 0.984
Elevation of the thinnest point from the 8 mm posterior BFTE	0.933	0.0264	0.885 to 0.965
Maximum elevation from the 8 mm posterior BFTE	0.806	0.0342	0.739 to 0.861
Minimum elevation from the 8 mm posterior BFTE	0.923	0.022	0.873 to 0.958
Maximum minus Minimum elevation from the 8 mm posterior BFTE	0.912	0.0225	0.861 to 0.950
Elevation of the thinnest point from the 9 mm anterior BFS	0.979	0.0118	0.945 to 0.995
Elevation of the thinnest point from the 9 mm posterior BFS	0.977	0.0098	0.942 to 0.994
Maximum elevation from the 9 mm posterior BFS	0.772	0.0364	0.703 to 0.832
Minimum elevation from the 9 mm posterior BFS	0.865	0.0294	0.805 to 0.912
Maximum minus minimum elevation from the 9 mm posterior BFS	0.832	0.0324	0.768 to 0.884
Elevation of the thinnest point from the 9 mm anterior BFS minus that of posterior BFS	0.952	0.0171	0.909 to 0.979
Elevation of the thinnest point from the 9 mm posterior BFTE	0.904	0.0309	0.851 to 0.943
Maximum elevation from the 9 mm posterior BFTE	0.671	0.0451	0.597 to 0.740
Minimum elevation from the 9 mm posterior BFTE	0.934	0.0204	0.886 to 0.966
Maximum minus minimum elevation from the 9 mm posterior BFTE	0.887	0.0264	0.831 to 0.930

*Pachymetry indices*			

Apex thickness	0.897	0.0268	0.842 to 0.938
Central corneal thickness	0.878	0.0288	0.820 to 0.922
Thinnest point thickness	0.915	0.0241	0.863 to 0.952
PPI-Min	0.939	0.0208	0.893 to 0.969
PPI-Avg	0.978	0.00973	0.944 to 0.994
PPI-Max	0.987	0.00563	0.958 to 0.998
PPI-Max minus PPI-Min	0.903	0.0246	0.850 to 0.943
ART-Min	0.949	0.0183	0.905 to 0.976
ART-Avg	0.976	0.0101	0.942 to 0.993
ART-Max	0.987	0.00605	0.957 to 0.998
Horizontal decentration of the thinnest point from the apex	0.558	0.0444	0.481 to 0.633
Vertical decentration of the thinnest point from the apex	0.737	0.0397	0.666 to 0.801
Diagonal decentration of the thinnest point from the apex	0.686	0.0403	0.612 to 0.754

AUROC: area under the receiver operating characteristic curve. SEM: standard error of the mean. 95% CI: 95% confidence interval of the AUROC. BFS: best-fit sphere, BFTE: best-fit toric ellipsoid, PPI-Min: Pachymetry Progression Index-Minimum, PPI-Avg: Pachymetry Progression Index-Average, PPI-Max: Pachymetry Progression Index-Maximum, ART-Min: Ambrósio's Relational Thickness-Minimum, ART-Avg: Ambrósio's Relational Thickness-Average, and ART-Max: Ambrósio's Relational Thickness-Maximum.

**Table 3:** The indices with highest AUROC.

Indices	AUROC	95% CI	Criterion	Sensitivity	Specificity	LR+	LR−	AUROC compared to that of PPI-Max
PPI-Max	0.987	0.958 to 0.998	>1.4	91.78	98.06	47.27	0.084	
ART-Max	0.987	0.957 to 0.998	≤412	97.26	93.2	14.31	0.029	*P* = 0.880
Elevation of the thinnest point from the 9 mm anterior BFS	0.979	0.945 to 0.995	>7	95.89	92.23	12.35	0.045	*P* = 0.501
Elevation of the thinnest point from the 8 mm anterior BFS	0.968	0.930 to 0.989	>5	91.78	96.12	23.63	0.086	*P* = 0.288
Elevation of the thinnest point from the 8 mm posterior BFS	0.979	0.945 to 0.995	>20	86.3	100	N/A	0.14	*P* = 0.229
Elevation of the thinnest point from the 7 mm posterior BFS	0.971	0.934 to 0.990	>10	87.67	100	N/A	0.12	*P* = 0.217
Elevation of the thinnest point from the 9 mm posterior BFS	0.977	0.942 to 0.994	>22	89.04	96.12	22.93	0.11	*P* = 0.199
PPI-Avg	0.978	0.944 to 0.994	>1.1	87.67	98.06	45.15	0.13	*P* = 0.147
Elevation of the thinnest point from the 7 mm posterior BFTE	0.953	0.910 to 0.979	>10	87.67	99.03	90.3	0.12	*P* = 0.104
ART-Avg	0.976	0.942 to 0.993	≤496	94.52	94.17	16.23	0.058	*P* = 0.089
All other tested indices								*P* < 0.05

AUROC: area under the receiver operating characteristic curve. 95% CI: 95% confidence interval of the AUROC. LR+: likelihood ratio of positive results. LR−: likelihood ratio of negative results. *P* from PPI-Max: probability of chance that the AUROC is less than that of PPI-Max. PPI-Max: Pachymetry Progression Index-Maximum, ART-Max: Ambrósio's Relational Thickness-Maximum, BFS: best-fit sphere, PPI-Avg: Pachymetry Progression Index-Average, BFTE: best-fit toric ellipsoid, and ART-Avg: Ambrósio's Relational Thickness-Average.

**Table 4:** Scheimpflug imaging indices differentiating between GROUP 2a KC and normal corneas.

Indices	AUROC	SEM	*95% CI*	*P* value
PPI-Max	0.690	0.038	0.615 to 0.765	0.018
ART-Max	0.690	0.038	0.615 to 0.764	0.019
PPI-Max minus PPI-Min	0.687	0.055	0.579 to 0.796	0.020
Central corneal thickness	0.683	0.048	0.590 to 0.776	0.023
Diagonal decentration of the thinnest point from the apex	0.674	0.062	0.552 to 0.795	0.031

AUROC: area under the receiver operating characteristic curve, SEM: standard error of the mean, 95% CI: 95% confidence interval of the AUROC, PPI-Max: Pachymetry Progression Index-Maximum, ART-Max: Ambrósio's Relational Thickness-Maximum, and PPI-Min: Pachymetry Progression Index-Minimum.
